# Carnosic Acid against Lung Cancer: Induction of Autophagy and Activation of Sestrin-2/LKB1/AMPK Signalling

**DOI:** 10.3390/ijms25041950

**Published:** 2024-02-06

**Authors:** Eric J. O’Neill, Newman Siu Kwan Sze, Rebecca E. K. MacPherson, Evangelia Tsiani

**Affiliations:** Department of Health Sciences, Faculty of Applied Health Sciences, Brock University, St. Catharines, ON L2S 3A1, Canada; eo15nv@brocku.ca (E.J.O.); nsze@brocku.ca (N.S.K.S.); rmacpherson@brocku.ca (R.E.K.M.)

**Keywords:** NSCLC, carnosic acid, autophagy, apoptosis, polyphenols, lung cancer, AMPK, sestrin-2, LKB1

## Abstract

Non-small cell lung cancer (NSCLC) represents 80% of all lung cancer cases and is characterized by low survival rates due to chemotherapy and radiation resistance. Novel treatment strategies for NSCLC are urgently needed. Liver kinase B1 (LKB1), a tumor suppressor prevalently mutated in NSCLC, activates AMP-activated protein kinase (AMPK) which in turn inhibits mammalian target of rapamycin complex 1 (mTORC1) and activates unc-51 like autophagy activating kinase 1 (ULK1) to promote autophagy. Sestrin-2 is a stress-induced protein that enhances LKB1-dependent activation of AMPK, functioning as a tumor suppressor in NSCLC. In previous studies, rosemary (*Rosmarinus officinalis*) extract (RE) activated the AMPK pathway while inhibiting mTORC1 to suppress proliferation, survival, and migration, leading to the apoptosis of NSCLC cells. In the present study, we investigated the anticancer potential of carnosic acid (CA), a bioactive polyphenolic diterpene compound found in RE. The treatment of H1299 and H460 NSCLC cells with CA resulted in concentration and time-dependent inhibition of cell proliferation assessed with crystal violet staining and ^3^H-thymidine incorporation, and concentration-dependent inhibition of survival, assessed using a colony formation assay. Additionally, CA induced apoptosis of H1299 cells as indicated by decreased B-cell lymphoma 2 (Bcl-2) levels, increased cleaved caspase-3, -7, poly (ADP-ribose) polymerase (PARP), Bcl-2-associated X protein (BAX) levels, and increased nuclear condensation. These antiproliferative and proapoptotic effects coincided with the upregulation of sestrin-2 and the phosphorylation/activation of LKB1 and AMPK. Downstream of AMPK signaling, CA increased levels of autophagy marker light chain 3 (LC3), an established marker of autophagy; inhibiting autophagy with 3-methyladenine (3MA) blocked the antiproliferative effect of CA. Overall, these data indicate that CA can inhibit NSCLC cell viability and that the underlying mechanism of action of CA involves the induction of autophagy through a Sestrin-2/LKB1/AMPK signaling cascade. Future experiments will use siRNA and small molecule inhibitors to better elucidate the role of these signaling molecules in the mechanism of action of CA as well as tumor xenograft models to assess the anticancer properties of CA in vivo.

## 1. Introduction

Cancer is a leading cause of death globally, second only to cardiovascular disease, which made up nearly 10 million deaths in 2020 [1]. Lung cancer is the leading cause of cancer mortality globally, comprising approximately 18% of cancer deaths [2]. Around 80–85% of lung cancer cases are categorized as non-small cell lung cancer (NSCLC) which is further divided into large cell carcinoma, squamous cell carcinoma, and adenocarcinoma accounting for 10–15%, 25%, and 40% of all lung cancer cases, respectively [3]. The treatment for NSCLC typically involves surgery if the primary tumor is resectable, combined with radiation, chemotherapy, immunotherapy, or targeted therapies [4]. Despite treatment options, roughly 72% of NSCLC patients will not survive beyond 5 years underscoring a need for research into novel treatments [5].

A key feature of NSCLC is dysregulated signal transduction cascades that ultimately contribute to uncontrolled cell proliferation, the evasion of apoptosis, and metastasis. Two common pathways implicated in NSCLC are the Ras-Raf-MEK-extracellular signal-regulated kinase (ERK) cascade and the phosphoinositide 3-kinase (PI3K)-Akt-mammalian target of rapamycin (mTOR) cascade [6]. Epidermal growth factor receptor (EGFR) is frequently overexpressed in NSCLC leading to the increased activation of Ras-Raf-MEK-ERK signaling. Additionally, Ras and Raf mutations can further contribute to increased ERK activation and enhanced cell survival and proliferation. Activated Ras also binds and activates PI3K leading to the activation of Akt-mTORC1 signaling resulting in increased protein synthesis and proliferation, and the inhibition of autophagy [6]. *PIK3CA* mutations that yield a constitutively active form of PI3K are present in some NSCLC cases, which also contributes to enhanced Akt-mTORC1 signaling [6]. Compounds capable of targeting these dysregulated cascades such as the EGFR tyrosine kinase inhibitor gefitinib are often used to treat NSCLC; however, acquired therapy-resistance in NSCLC is a significant problem [7].

Many chemotherapy drugs used to treat NSCLC are derived from plants including etoposide from wild mandrake (*Podophyllum peltatum*) [8] and paclitaxel from pacific yew (*Taxus brevifolia*) bark [9]. Rosemary (*Rosmarinus officinalis*) is a herb with reported antioxidant [10], antimicrobial [11], antidiabetic [12,13], and anticancer properties [14]. Several studies have shown that rosemary extract (RE) and its polyphenolic compounds—carnosol, carnosic acid (CA), and rosmarinic acid—reduce the proliferation and survival of cancer cells and inhibit tumor progression in animal models [14,15]. In recent studies by our group, RE inhibited the proliferation and survival, and induced the apoptosis of lung [16,17], breast [18], and prostate [19] cancer cells. In NSCLC cells, RE significantly increased AMPK signaling, but the active pharmaceutical ingredient responsible and the mechanism leading to AMPK activation has not been explored [17].

AMP-activated protein kinase (AMPK) is an energy sensor whose activation is associated with increased survival in NSCLC patients [20]. Additionally, the anticancer effects of several polyphenols are associated with AMPK activation, including quercetin [21], resveratrol [22], and epigallocatechin-3-gallate (EGCG) [23]. Research has indicated that RE and RE polyphenols trigger apoptosis and cell cycle arrest in a variety of cancer cells as well as increased activation of AMPK [14,15]. Sestrin-2 is a stress-induced protein that leads to the activation of AMPK signaling during conditions of nutrient deficiency, DNA damage, oxidative stress, or hypoxia [24,25]. Liver kinase B1 (LKB1) is a tumor suppressor and upstream kinase of the AMPK pathway that is mutated in 39% of NSCLC cell lines and 34% of human lung adenocarcinoma specimens [26,27,28]. Sestrin-2 has been shown to increase the LKB1-dependent activation of AMPK [29]. The phosphorylation of LKB1 at the Ser^428^ residue mediates its association with, and the activation of AMPK [30]. When activated, AMPK activates fatty acid catabolism and inhibits fatty acid synthesis. Additionally, activated AMPK inhibits mTOR signaling and protein synthesis, and activates autophagy. Autophagy is a process of cellular self-degradation initiated in response to stressors such as nutrient deprivation that have been reported to have conflicting roles in cancer [31]. While low levels of autophagy are thought to aid cancer progression, excessive levels of autophagy, such as those induced by curcumin [32] or apatinib [33], damage cells and induce apoptosis.

In the current study, we focused on CA, a bioactive benzenediol abietane diterpene, examined its effects in lung cancer cells, and investigated the role of upstream and downstream AMPK signaling in its mechanism of action.

## 2. Results

### 2.1. Carnosic Acid Inhibits Proliferation and Survival of NSCLC Cells

**Figure 1:**
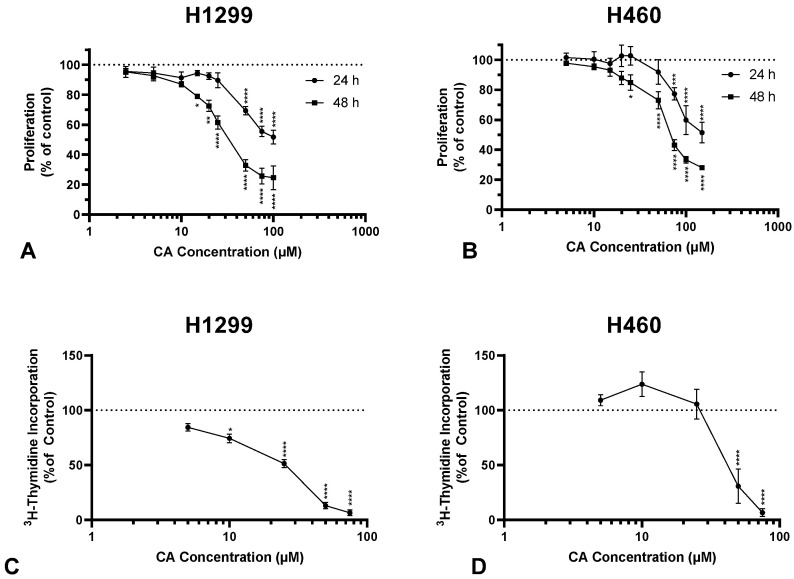
Carnosic acid (CA) inhibits proliferation of NSCLC cells. Proliferation was assessed using a crystal violet assay (**A**,**B**) and a ^3^H-thymidine incorporation assay (**C**,**D**). H1299 and H460 cells were seeded and then treated the following day with CA (**A**: 2.5–100 µM; **B**: 5–150 µM) for 24 or 48 h, fixed, stained with crystal violet, crystal violet dye solubilized, and then absorbance measured at 590 nm and expressed as a percentage of control; data are the mean ± SEM for three individual experiments (**A**). Cells were treated with increasing concentrations of CA (5–75 µM) and exposed to ^3^H-thymidine for 24 h (**B**). Cells were lysed and radioactivity measured using liquid scintillation counting. Data are the mean ± SEM for four individual experiments expressed as a percentage of control (**B**). * *p* < 0.05, ** *p* < 0.01, *** *p* < 0.001, **** *p* < 0.0001, compared to control (**A**,**B**).

The accumulation of mutations in cancer cells results in unregulated cell proliferation. The proliferation of H1299 and H460 cells in the presence of CA was assessed using crystal violet and thymidine incorporation assays. CA caused concentration and time-dependent inhibition of H1299 cell proliferation with an IC_50_ of 47.3 µM and 27.1 µM for 24 h and 48 h, respectively (Figure 1A). Proliferating cells incorporate thymidine as they synthesize new DNA for mitosis. CA inhibited ^3^H-thymidine incorporation in H1299 cells in a concentration-dependent manner with significant inhibition observed with 10 µM (74.44 ± 3.93% of control, *p* < 0.05), maximum inhibition with 50 µM (13.11 ± 2.92% of control, *p* < 0.0001), and an IC_50_ of 41.09 µM (Figure 1C).

In addition, CA treatment dose-dependently inhibited the proliferation of H460 cells, with an IC_50_ of 89.6 µM and 67 µM for 24 h and 48 h, respectively (Figure 1B). The maximum inhibition of ^3^H-thymidine incorporation in H460 cells was observed with 50 µM CA (30.77 ± 15.73% of control, *p* < 0.0001; Figure 1D).

**Figure 2:**
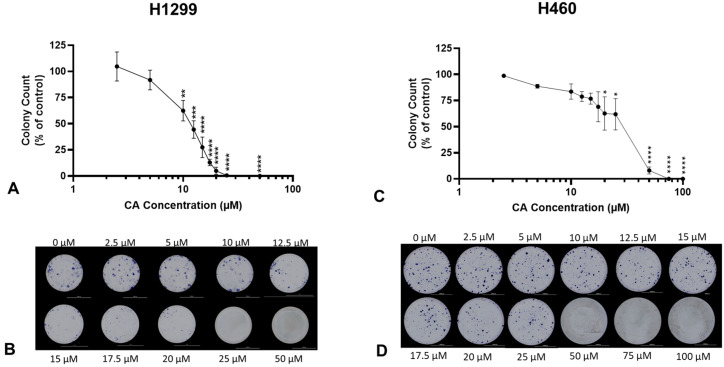
Carnosic acid (CA) inhibits survival of NSCLC cells. H1299 (**A**,**B**) and H460 (**C**,**D**) cells were seeded at low density and treated the following day with increasing (**A**: 2.5–50 µM; **C**: 2.5–100 µM) concentrations of CA for 7 days to assess colony formation. Whole-well representative images were taken using a BioTek Cytation5 plate reader and BioTek Gen5 software (**B**,**D**). Following treatment, colonies greater than 50 cells were counted and expressed as a percentage of control; data are the mean ± SEM for three individual experiments. * *p* < 0.05, ** *p* < 0.01, *** *p* < 0.001, **** *p* < 0.0001, compared to control.

Colony formation is a characteristic of cancer cells grown in vitro and is representative of an individual cell’s ability to survive in isolation to form a new clonal subpopulation. A clonogenic survival assay [18] was used to assess the survival of NSCLC cells under CA treatment. Cells were treated with increasing concentrations of CA for 7 days. Following treatment, the cells were fixed and stained, and colonies greater than 50 cells were counted. CA caused a concentration-dependent inhibition of survival in both H1299 and H460 cells. Significant inhibition of H1299 colony formation was observed with concentrations of 10 µM (62.4 ± 9.8% of control, *p* < 0.01; Figure 2A) or greater and with IC_50_ of 11.7 µM. CA concentrations of 25 and 50 µM completely inhibited H1299 cell survival (25 µM: 0.5 ± 0.5% of control, *p* < 0.001; Figure 2A). Significant inhibition of H460 colony formation was observed with concentrations of 20 µM (62.65 ± 15.98% of control, *p* < 0.01; Figure 2C) or greater and with an IC_50_ of 29.7 µM.

### 2.2. Carnosic Acid Induces Apoptosis of H1299 NSCLC Cells

**Figure 3:**
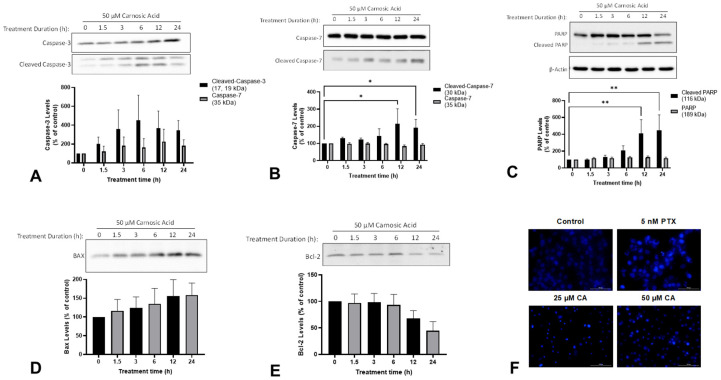
Carnosic acid (CA) induces apoptosis of H1299 NSCLC cells. Whole cell lysates were prepared from H1299 cells treated without (control) or with 50 µM CA for 1.5–24 h. Cell lysates (20 µg) were resolved by SDS-PAGE and immunoblotted with specific antibodies against caspase-3 (**A**) caspase-7 (**B**), PARP (**C**), BAX (**D**), Bcl-2 (**E**), or β-actin (**C**). Upper panel: A representative immunoblot is shown. Lower panel: The densitometry of the bands, expressed in arbitrary units was measured using ImageJ 1.54g software. H1299 cells were treated for 24 h with 5 nM paclitaxel (PTX), 25 µM CA, or 50 µM CA, and nuclear morphology was assessed qualitatively using Hoechst 33342 and a BioTek cytation 5 plate reader (**F**). The data are expressed as percent of control and are the mean ± SEM of 3–6 separate experiments. * *p*< 0.05, ** *p* < 0.01, compared to control.

The evasion of apoptosis is another characteristic of cancer cells that contributes to increased proliferation and survival. During apoptosis, proapoptotic Bcl-2-associated X protein (BAX) neutralizes antiapoptotic B-cell lymphoma 2 (Bcl-2) leading to the release of apoptogenic factors from mitochondria. These factors lead to activation of caspase-3 and caspase-7 which promote apoptosis through proteolytic cleavage and the inactivation of proteins necessary for survival such as poly (ADP-ribose) polymerase (PARP). The treatment of H1299 cells with 50 µM CA for 12 h or 24 h significantly increased the levels of cleaved caspase-7 (12 h: 214.8 ± 87.3%, *p* < 0.05; 24 h: 191.7 ± 48.0%, *p* < 0.05; Figure 3A) and cleaved PARP (12 h: 410.3 ± 163.9%, *p* < 0.01; 24 h: 447.9 ± 183.5%, *p* < 0.01; Figure 3B). Additionally, there was a trend toward increased levels of cleaved caspase-3 and BAX, and decreased levels of Bcl-2 (Figure 3A,D,E).

Apart from examining the aforementioned apoptosis markers, we also examined morphological changes in the cells. During apoptosis, cells undergo a process of controlled disassembly that includes nuclear condensation and nuclear fragmentation. The treatment of H1299 cells with CA resulted in substantial changes in nuclear morphology consistent with apoptosis (i.e., nuclear fragmentation) when compared to untreated cells. Specifically, CA-treated cells had noticeably smaller nuclei which is consistent with the nuclear condensation that occurs during apoptosis (Figure 3F). The treatment of the cells with 5 nM paclitaxel—a chemotherapy drug used in the treatment of NSCLC—resulted in the same changes in nuclear morphology.

### 2.3. Carnosic Acid Activates Sestrin-2/LKB1/AMPK Signalling in H1299 NSCLC Cells

**Figure 4:**
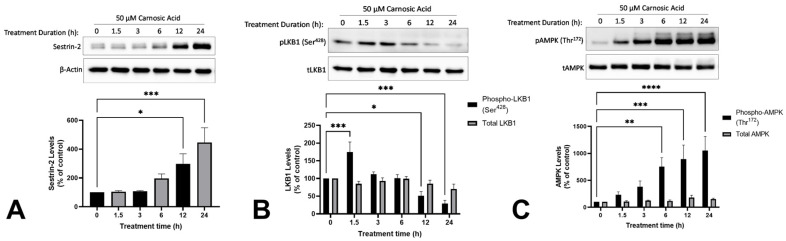
Carnosic acid (CA) increases Sestrin-2 (**A**), phospho-LKB1 (**B**), and phospho-AMPK (**C**) in H1299 NSCLC cells. Whole cell lysates were prepared from H1299 cells treated without (control) or with 50 µM CA for 1.5–24 h. Cell lysates (20 µg) were resolved by SDS-PAGE and immunoblotted with specific antibodies against sestrin-2 (**A**), β-actin (**A**), total and phospho-LKB1 (Ser^428^; **B**), or total and phospho-AMPK (Thr^172^; **C**). Upper panel: A representative immunoblot is shown. Lower panel: The densitometry of the bands, expressed in arbitrary units was measured using ImageJ 1.54 g software. The data are expressed as percent of control and are the mean ± SEM of six separate experiments. * *p* < 0.05, ** *p* < 0.01, *** *p* < 0.001, **** *p* < 0.0001, compared to control.

Treatment of H1299 NSCLC cells with 50 µM CA caused a time-dependent increase in levels of sestrin-2 (12 h: 297.9 ± 70.0%, *p* < 0.05; 24 h: 446.1 ± 102.2%, *p* < 0.001; Figure 4A). Treatment of H1299 NSCLC cells with CA resulted in a significant increase in the level of phosphorylated LKB1 after 1.5 h (175.0 ± 28.3%, *p* < 0.001). The levels of phosphorylated LKB1 decreased over time, returning to approximately basal levels at the 3 h (112.7 ± 6.1%, *p* = 0.9028) and 6 h (100.8 ± 10.2%, *p* > 0.9999) timepoints, and decreasing below basal at the 12 h (51.4 ± 11.7%, *p* < 0.05) and 24 h (28.9 ± 9.7%, *p* < 0.001) timepoints (Figure 4B). The treatment of H1299 NSCLC cells with CA caused a time-dependent increase in the levels of phosphorylated AMPK (Figure 4C). A significant increase was observed with treatment times of 6 h and above, with a tenfold increase at 24 h (1051.8 ± 262.7%, *p* < 0.0001; Figure 4C).

### 2.4. Carnosic Acid Activates Autophagy Signalling in H1299 NSCLC Cells

**Figure 5:**
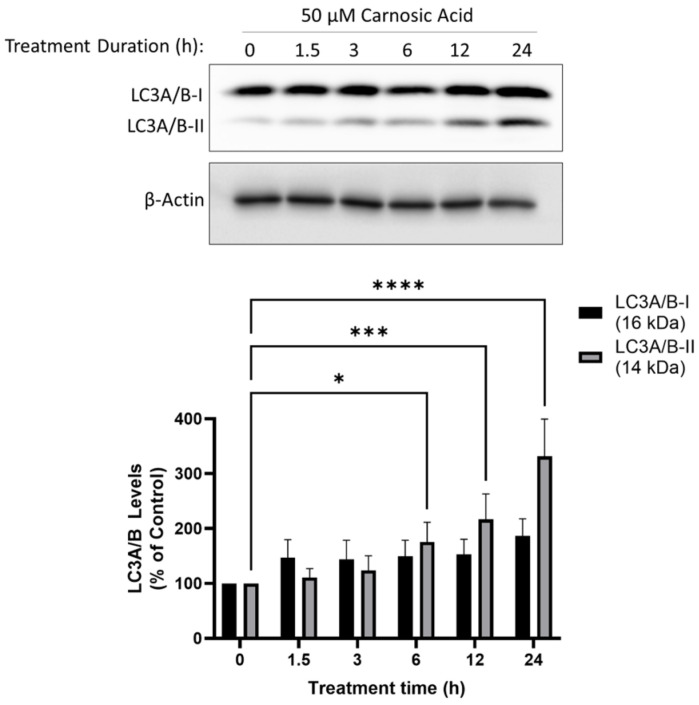
Carnosic acid (CA) induces autophagy in H1299 NSCLC cells. Whole cell lysates were prepared from H1299 cells treated without (control) or with 50 µM CA for 1.5–24 h. Cell lysates (20 µg) were resolved by SDS-PAGE and immunoblotted with specific antibodies against LC3A/B or β-actin. **Upper panel**: A representative immunoblot is shown. **Lower panel**: The densitometry of the bands, expressed in arbitrary units was measured using ImageJ 1.54g software. The data are expressed as percent of control and are the mean ± SEM of six separate experiments. * *p*< 0.05, *** *p* < 0.001, **** *p* < 0.0001, compared to control.

LC3-I is converted to LC3-II during the later stages of autophagy and is used as a marker of autophagy. The treatment of H1299 cells with CA resulted in a time-dependent increase in levels of LC3-II with significance at 6 h (175.5 ± 35.9%, *p* < 0.05), 12 h (216.8 ± 46.3%, *p* < 0.001), and 24 h (331.4 ± 68.0%, *p* < 0.0001; Figure 5).

**Figure 6:**
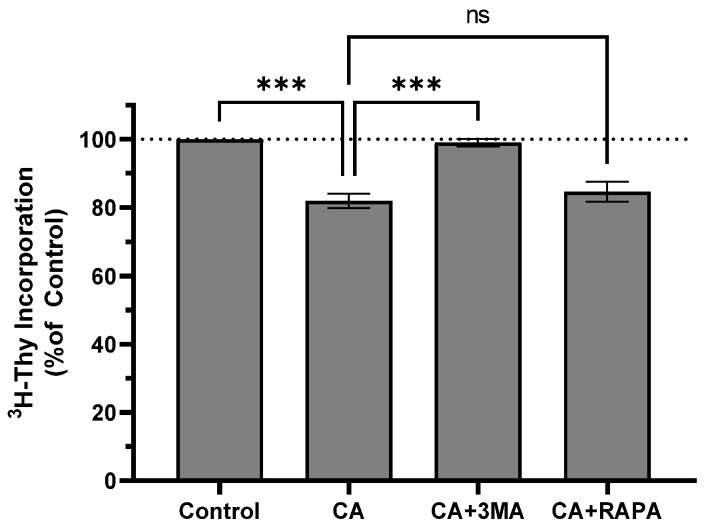
Autophagy inhibitor 3MA attenuates inhibitory effect of CA on thymidine incorporation. H1299 cells were pretreated for 1 h with autophagy inhibitor 3-methyladenine (3MA; 5 mM) or rapamycin (RAPA; 200 nM) followed by exposure to 10 µM carnosic acid (CA) for an additional 24 h. Cells were exposed to ^3^H-thymidine for 24 h. Cells were lysed, and radioactivity measured using liquid scintillation counting. Data are the mean ± SEM for three individual experiments expressed as a percentage of control. *** *p* < 0.001; ns, not significant.

To determine whether CA-induced autophagy was cytoprotective or antiproliferative, cells were treated with 10 µM CA in the presence of the autophagy inhibitor 3-methyladenine (3MA) or the autophagy activator rapamycin (RAPA). CA alone decreased thymidine incorporation to 82.00 ± 2.08% of control (*p* < 0.01 vs. control), and pre-treatment with 3MA blocked the effect of CA (99.00 ± 1.16% of control; *p* < 0.01 vs. CA). Pre-treatment with RAPA did not alter the effect of CA (84.67 ± 2.91% of control, *p* = 0.7187 vs. control; Figure 6). 3MA or RAPA alone did not affect ^3^H-thymidine incorporation (Figure S1).

## 3. Discussion

In the present study, we observed a significant inhibition of NSCLC cell proliferation with CA treatment. While several studies have indicated that CA possesses anticancer properties, its specific impact on NSCLC remains incompletely understood. Zhao et al. [34] examined the effects of CA in A549 NSCLC cells and found a dose-dependent inhibition of proliferation and survival, the induction of apoptosis, and the inhibition of migration and invasion. The effects of CA coincided with decreased levels of matrix metalloproteinase (MMP)-9, and inhibition of PI3K/Akt/mTOR signaling [34]. In the current study, higher concentrations of CA were required to inhibit the proliferation and ^3^H-thymidine incorporation in H460 cells compared to H1299 cells. This lower sensitivity of H460 cells to CA treatment may be due to their lack of the tumor suppressor LKB1 and is in agreement with the findings of Corveloni et al. [35] showing that similar concentrations of CA are required to inhibit H460 cells.

Although few studies have examined the effects of CA in NSCLC (and none in H1299), there are several studies showing antiproliferative effects in other types of cancer. Yesil-Celiktas et al. [36] assessed the antiproliferative effects of CA across a panel of cancer cell lines (K-562, MCF-7, Hep-3B, PC-3, DU-14, and MDA-MB-231) and found potent antiproliferative effects ranging from 13 to 30% of control with 19 µM CA treatment for 48 h. CA treatment for 24–72 h inhibited the proliferation of AGS and MKN-45 gastric cancer cells with the IC_50_ ranging from 53 to 72 µM [37]. Similarly, 24 h CA treatment inhibited the proliferation of colorectal cancer cells (caco-2, HT29 and LoVo) with the IC_50_ ranging from 24 to 96 µM [38]. CA caused a dose-dependent inhibition of CaSki and SiHa cervical cancer cell proliferation and colony formation at concentrations over 10 µM [39]. Our findings in H1299 confirm the antiproliferative effects of CA in the micromolar range (Figure 1) and provide novel information related to CA treatment in a human NSCLC cell line.

Apoptosis is controlled cell death in response to damage that plays an essential role in tissue homeostasis [40]. Apoptosis is triggered when a stress signal such as irradiation or increased reactive oxygen species (ROS) leads to the activation of proapoptotic members of the Bcl-2 family, such as Bax, which neutralize antiapoptotic proteins like Bcl-2 leading to disruption of mitochondrial membrane permeability and release of apoptogenic factors such as cytochrome-c [41]. Cytochrome-c in turn triggers the formation of the apoptosome which recruits the initiator pro-caspase-9 causing its autoactivation. The activation of initiator caspases activates downstream executor caspases-3, -6, and -7 which cleave and inactivate the substrates necessary for proliferation such as the DNA repair protein PARP [42]. In the current study, we observed a time-dependent induction of apoptosis as indicated by significantly increased levels of cleaved caspase-7 and cleaved PARP (Figure 3B,C). We also saw a trend towards increased levels of the apoptotic markers cleaved caspase-3 and Bax, and decreased levels of the antiapoptotic protein Bcl-2. Additionally, we observed nuclear condensation that is consistent with apoptosis (Figure 3F). Our findings are in agreement with microscopic evidence of CA-induced apoptosis observed in A549 NSCLC cells [34]. CA-induced apoptosis has also been observed in gastric [37], cervical [39], and colorectal [38] cancer cells including increased caspase-7 and PARP cleavage, and changes in nuclear morphology.

Some studies suggest that AMPK is a potential target for cancer treatment and prevention. The expression of phosphorylated AMPK in histological samples from lung adenocarcinoma patients was associated with higher survival compared to patients who tested negative for phosphorylated AMPK [20]. In a previous study, we observed AMPK activation in H1299 cells with RE treatment, but the signaling upstream and downstream of AMPK was not investigated extensively [17]. Here, we hypothesized that CA activates AMPK and we wanted to investigate potential upstream signaling molecules: sestrin-2 and LKB1. The induction of sestrin-2 has been shown to have anticancer effects in NSCLC cells [43]. The knockdown of orphan nuclear receptor TR3 in A549, H460, and H1299 cells led to the inhibition of proliferation and the induction of apoptosis which was attributed to the induction of sestrin-2 and the activation of AMPK [43]. LKB1 is an upstream kinase of AMPK and it has been shown that sestrin-2 acts as a scaffold to facilitate the interaction between LKB1 and AMPK [29,44,45]

Under physiological conditions, sestrin-2 expression is induced by DNA damage in a p53-dependent manner or by ROS independent of p53. In the current study, we report a time-dependent increase in sestrin-2 expression (Figure 4A). The treatment of colon cancer cells (HCT116 and HT-29) with quercetin also increases sestrin-2 expression and induces apoptosis [46]. These effects are ROS-induced, p53-independent, as well as sestrin-2 and AMPK-dependent [46]. H1299 cells lack the tumor suppressor p53 and have low basal levels of sestrin-2. It is possible that CA exhibits antitumor effects in H1299 (which lack p53 expression) cells by inducing sestrin-2 expression independent of p53, similar to the effects of quercetin in colon cancer cells.

Here, we saw an increase in Ser^428^ phosphorylation of LKB1 with acute LKB1 treatment (Figure 4B). LKB1 is implicated as an upstream kinase required for the anticancer effects of several natural products. The polyphenol honokiol increased LKB1 expression in breast cancer cells (MCF7 and MDA-MB-231) and LKB1 knockdown blocked the anticancer effects [47]. Similarly, the anticancer effects of the polyphenol resveratrol in leukemic cells (HL-60) were dependent on autophagy activated via LKB1-AMPK signaling [48]. Curcumin exhibited anticancer effects in colon cancer cells (Caco-2) by increasing Ser^428^ phosphorylation of LKB1 and the subsequent activation of AMPK [49]. In a xenograft model of breast cancer, mango (*Mangifera indica* L.) polyphenols decreased tumor weight and volume, and this was associated with increased sestrin-2, LKB1, and AMPK levels [50]. Taken together, our data indicate that sestrin-2/LKB1/AMPK signaling may be a possible mechanism contributing to the antiproliferative and proapoptotic effects of CA, similar to other polyphenols, and this could explain why CA was less effective in the LKB1-null H460 cell line.

In the current study, CA treatment resulted in a time-dependent increase in LC3-II levels consistent with induction of autophagy (Figure 5). CA-induced autophagy could be a cytoprotective mechanism in response to cellular stress caused by CA. This was the case with the naturally sourced anticancer compounds polyphyllin II and pyoluteorin whose anticancer effects were enhanced by autophagy inhibition with 3MA [51,52]. Alternatively, CA-induced autophagy could be contributing to cell death, as was the case with quercetin. Quercetin treatment of H1299 resulted in the inhibition of proliferation, AMPK activation, and the induction of autophagy and autophagy inhibition with 3MA-inhibited quercetin-induced apoptosis [21]. To determine whether autophagy was contributing to cell death or cell survival, we treated H1299 cells with a sub-maximal dose (10 µM) of CA in the presence of the autophagy inhibitor 3MA or the autophagy activator RAPA. We hypothesized that if the CA-induced autophagy was cytoprotective, then 3MA should enhance the antiproliferative effect of CA, and RAPA would attenuate the antiproliferative effect of CA. We found that 3MA blocked the effect of CA while RAPA had no effect. This suggests that in our model, autophagy is contributing to the CA-induced inhibition of proliferation.

Other studies also suggest that sestrin-2-mediated autophagy has antiproliferative effects in some cancer cells. ChlA-F, a derivative of cheliensisin A [53], and isorhapontigenin, a derivative of stilbene [54], inhibit the anchorage-independent growth of bladder cancer cells by inducing sestrin-2-dependent autophagy. In colorectal cancer cells, the combination of oxaliplatin and docosahexaenoic acid caused autophagic cell death mediated by endoplasmic reticulum stress and upregulation of sestrin-2 [55]. In hepatocellular carcinoma cells (HepG2 and PLC/PRF/5), fangchinoline inhibited cell proliferation survival, and induced Sestirn-2/AMPK mediated autophagic cell death [56]. The effects of tanshinone IIA, a diterpenoid naphthoquinone against osteosarcoma cells, also involved sestrin-2-mediated autophagy [57]. CA may act through similar mechanisms to induce autophagy and cell death in lung cancer cells.

## 4. Materials and Methods

### 4.1. Materials

Human H1299 cells were obtained from the American Type Culture Collection (ATCC). Cell culture (RPMI) media, fetal bovine serum (FBS), trypsin, antibiotic, and Hoechst 33342 were from Thermo Fisher Scientific (Burlington, ON, Canada). Antibodies against caspase-3 (cat. No. 9662), caspase-7 (cat. No. 9494), PARP (cat. No. 9542), BAX (cat. No. 2772), Bcl-2 (cat. No. 2872), β-actin (cat. No. 4967), sestrin-2 (cat. No. 8487), pLKB1, LKB1 (cat. No. 3047), pAMPK (cat. No. 2535), AMPK (cat. No. 5831), and LC3 (cat. No. 12741) were purchased from Cell Signaling Technology via New England Biolabs (Mississauga, ON, Canada). Bovine serum albumin (BSA), 10% formalin, dimethyl sulfoxide (DMSO), crystal violet stain, and paclitaxel were purchased from Millipore Sigma (Oakville, ON, Canada). Carnosic acid (purity 98.17%) and 3-methyladenine were purchased from Med Chem Express (Monmouth Junction, NJ, USA).

### 4.2. Cell Culture

H1299 and H460 cells were cultured in RPMI media supplemented with 10% (*v*/*v*) FBS and 1% (*v*/*v*) antibiotic-antimycotic in a humidified incubator at 37 °C with 5% CO_2_. A stock solution of 100 mM CA was prepared in DMSO and diluted to a working concentration of 1 mM with cell culture media. The final concentration and time of exposure are indicated in each figure.

### 4.3. Crystal Violet Proliferation Assay

The crystal violet cell proliferation assay was performed as described previously [17]. H1299 or H460 cells were seeded (1000 cells/well) in sextuplicate in 96-well plates and treated with the indicated concentration of CA for 24 or 48 h. Following treatment, cells were fixed with 10% formalin and stained with 0.5% (*w*/*v*) crystal violet. Crystal violet dye was solubilized, and absorbance was read at 570 nm using a KC4 plate reader (Bio-Tek, Winooski, VT, USA). The data are expressed as a percentage of the control.

### 4.4. [^3^H]-Thymidine Incorporation Assay

The [^3^H]-thymidine incorporation assay was performed as described previously [58]. Subconfluent H1299 was serum-deprived for 24 h and then treated with the indicated concentrations of CA followed by the addition of 10 μM [^3^H]-thymidine for 24 h. Following treatment, the media was removed, and the cells were rinsed three times with ice-cold HEPES-buffered saline (HBS). Unincorporated [^3^H]-thymidine was solubilized with 10% trichloroacetic acid (TCA) for 10 min at 4 °C. The TCA was aspirated, and the cells were rinsed twice with ice-cold HBS, followed by lysis with 0.05 N NaOH. The radioactivity of lysates was determined using liquid scintillation counting and data were expressed as a percentage of control.

### 4.5. Clonogenic Survival Assay

Clonogenic survival assays were performed as described previously [17]. H1299 or H460 cells were seeded in duplicate in 12-well plates (500 cells/well), allowed to adhere overnight, and exposed to media containing the indicated concentrations of CA for 7 days. Colonies were fixed with 10% formalin and stained with 0.5% (*w*/*v*) crystal violet; representative images were taken using a BioTek Cytation5 plate reader. Colonies (>50 cells) were counted, and the data were expressed as the surviving fraction compared to the control.

### 4.6. SDS-PAGE and Western Blotting

SDS-PAGE and Western blotting were performed as described previously [17]. Following treatment, cells were washed with ice-cold PBS and lysed with ice-cold RIPA buffer containing phosphatase and protease inhibitor. Lysate was combined with Laemmli sample buffer and boiled for 5 min. Proteins (20 µg) were separated using SDS-PAGE, transferred to a PVDF membrane, and incubated with the indicated antibodies. Low molecular weight targets (<40 kDa), mid-range targets (40–100 kDa), and high molecular weight targets (>100 kDa) were electrophoresed using hand-cast 12.5%, 10%, or 7.5% polyacrylamide gels, respectively. β-actin was used as a loading control. Blots were visualized using a BioRad chemidoc imager. Densitometric analysis was performed using Image J 1.54g software. The data are expressed as the mean ± SEM relative to the control.

### 4.7. Statistical Analysis

All data are expressed as the mean ± SEM for the indicated number of individual experiments. Analysis of variance (ANOVA) was performed using GraphPad Prism 8 software. All *p*-values ≤ 0.05 were considered significant. Any significant ANOVA result was followed with Dunnett’s post hoc test.

## 5. Conclusions

Previously, we have shown that rosemary (*Rosmarinus officinalis*) extract (RE) inhibits the proliferation, survival, and migration of H1299 NSCLC cells via a mechanism involving the activation of AMPK and the induction of apoptosis [17]. In the current study, we found that the treatment of H1299 and H460 NSCLC cells with CA (a RE polyphenolic diterpene) resulted in the inhibition of proliferation and survival. In H1299 cells, CA induced apoptosis and autophagy, and these effects correlated with increased levels of sestrin-2, phospho-LKB1, and phospho-AMPK. Inhibiting autophagy with 3MA blocked the antiproliferative effect of CA (see graphical abstract). Taken together, these results indicate that CA can induce the apoptosis of H1299 NSCLC cells through a mechanism involving sestrin-2/LKB1/AMPK signaling and the induction of autophagy. 

## Data Availability

Data are contained within the article and supplementary information.

## Supplementary Materials

The following supporting information can be downloaded at: https://www.mdpi.com/article/10.3390/ijms25041950/s1.
