# Supplementary material for: Carnosic Acid against Lung Cancer: Induction of Autophagy and Activation of Sestrin-2/LKB1/AMPK Signalling
Source: Int J Mol Sci. 2024 Feb 6;25(4):1950. doi: 10.3390/ijms25041950 (PMC10888478; doi:10.3390/ijms25041950)
Supplement: Supplementary file 1 [file ijms-25-01950-s001.zip › ijms-2792383-supplementary.pdf]

International Journal of *Molecular Sciences*

MDPI

Supplementary

Carnosic acid against lung cancer: induction of autophagy and activation of Sestrin-2/LKB1/AMPK signalling

Eric J. O'Neill [1], Newman Siu Kwan Sze[1], Rebecca E. K. MacPherson[1] and Evangelia Tsiani [1,*]

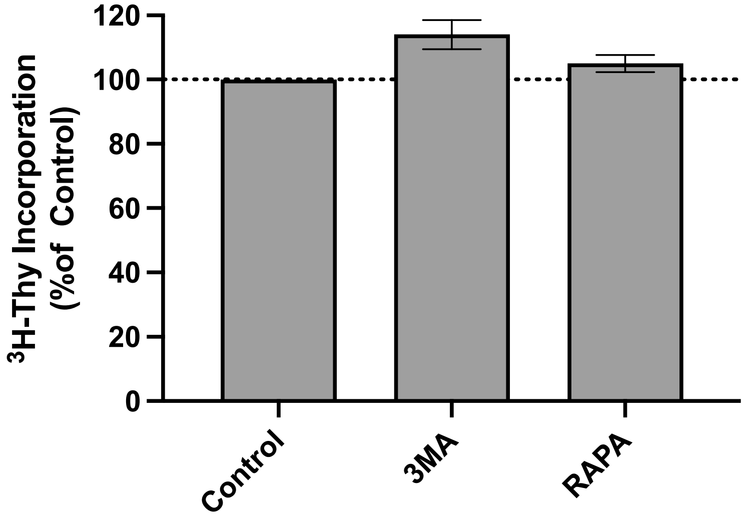

Figure S1. Effect of 3-methyladenine (3MA) and rapamycin (RAPA) on ^{3}H-thymidine incorporation in H1299 cells. H1299 cells were pretreated for 1 h with autophagy inhibitor 3-methyladenine (3MA; 5 mM) or rapamycin (RAPA; 200 nM) followed by exposure to ^{3}H-thymidine for 24 h. Cells were lysed, and radioactivity measured using liquid scintillation counting. Data are the mean ± SEM for 3 individual experiments expressed as a percentage of control.
